# Efficiency of a clinical prediction model for selective rapid testing in children with pharyngitis: A prospective, multicenter study

**DOI:** 10.1371/journal.pone.0172871

**Published:** 2017-02-24

**Authors:** Jérémie F. Cohen, Robert Cohen, Philippe Bidet, Annie Elbez, Corinne Levy, Patrick M. Bossuyt, Martin Chalumeau

**Affiliations:** 1 Department of General Pediatrics, Necker – Enfants malades hospital, Assistance Publique – Hôpitaux de Paris, Paris Descartes University, Paris, France; 2 Inserm UMR 1153, Obstetrical, Perinatal and Pediatric Epidemiology Research Team, Research Center for Epidemiology and Biostatistics Sorbonne Paris Cité (CRESS), Paris Descartes University, Paris, France; 3 Department of Clinical Epidemiology, Biostatistics and Bioinformatics, Academic Medical Centre, University of Amsterdam, Amsterdam, Netherlands; 4 Association Clinique et Thérapeutique Infantile du Val-de-Marne (ACTIV), Saint-Maur-des-Fossés, France; 5 Department of Microbiology, Centre Hospitalier Intercommunal de Créteil, Créteil, France; 6 Université Paris Est, IMRB-GRC GEMINI, Créteil, France; 7 Department of Microbiology, Robert-Debré Hospital, Assistance Publique – Hôpitaux de Paris, Paris Diderot University, Sorbonne Paris Cité, Paris, France; 8 Clinical Research Center, Centre Hospitalier Intercommunal de Créteil, Créteil, France; Centre Hospitalier Universitaire Vaudois, FRANCE

## Abstract

**Background:**

There is controversy whether physicians can rely on signs and symptoms to select children with pharyngitis who should undergo a rapid antigen detection test (RADT) for group A streptococcus (GAS). Our objective was to evaluate the efficiency of signs and symptoms in selectively testing children with pharyngitis.

**Materials and methods:**

In this multicenter, prospective, cross-sectional study, French primary care physicians collected clinical data and double throat swabs from 676 consecutive children with pharyngitis; the first swab was used for the RADT and the second was used for a throat culture (reference standard). We developed a logistic regression model combining signs and symptoms with GAS as the outcome. We then derived a model-based selective testing strategy, assuming that children with low and high calculated probability of GAS (<0.12 and >0.85) would be managed without the RADT. Main outcomes and measures were performance of the model (*c*-index and calibration) and efficiency of the model-based strategy (proportion of participants in whom RADT could be avoided).

**Results:**

Throat culture was positive for GAS in 280 participants (41.4%). Out of 17 candidate signs and symptoms, eight were retained in the prediction model. The model had an optimism-corrected *c*-index of 0.73; calibration of the model was good. With the model-based strategy, RADT could be avoided in 6.6% of participants (95% confidence interval 4.7% to 8.5%), as compared to a RADT-for-all strategy.

**Conclusions:**

This study demonstrated that relying on signs and symptoms for selectively testing children with pharyngitis is not efficient. We recommend using a RADT in all children with pharyngitis.

## Introduction

Group A streptococcus (GAS) is the most common bacterial cause of pharyngitis in children. About 37% of children with pharyngitis have GAS [1]; most of the remaining cases are of viral origin. Accurate diagnosis of pharyngitis is critical to ensure antibiotic treatment of GAS infections, and to limit antibiotic overuse. Because signs and symptoms of GAS and viral pharyngitis overlap broadly [2], the American Heart Association (AHA), the Infectious Diseases Society of America (IDSA), and the American Academy of Pediatrics (AAP) recommend microbiological confirmation of the presence of GAS (Table 1) [3–5].

**Table 1:** Key concepts in current North-American clinical practice guidelines for diagnosis of streptococcal pharyngitis, with corresponding quotes.

Concept	AHA [3]	IDSA [4]	AAP [5]
Microbiological testing is recommended because the clinical diagnosis of GAS is not accurate.	“Accurate differentiation of GAS pharyngitis from pharyngitis caused by other pathogens based on history and clinical findings is often difficult […]. Therefore, some form of microbiological confirmation […] is required for the diagnosis of GAS pharyngitis.”	“The clinical diagnosis of GAS pharyngitis cannot be made with certainty even by the most experienced physicians, and bacteriologic confirmation is required.”	“Diagnosis of GAS pharyngitis requires confirmation by rapid testing or culture.” “GAS should not be diagnosed in the absence of testing.”
Rapid tests should be used selectively in patients with signs and symptoms suggestive of GAS.	“When deciding whether to perform a microbiological test for a patient with acute pharyngitis, […] clinical and epidemiological findings […] need to be considered […]. If these findings are suggestive of GAS pharyngitis, then a throat culture or RADT should be performed to confirm the diagnosis.”	“GAS testing should be performed on selected patients with clinical symptoms and signs on physical examination that are suggestive of GAS.”	“Patients with 2 or more of the following features should undergo testing: (1) absence of cough, (2) presence of tonsillar exudates or swelling, (3) history of fever, (4) presence of swollen and tender anterior cervical lymph nodes, and (5) age younger than 15 years.“

Abbreviations: AHA, American Heart Association; IDSA, Infectious Diseases Society of America; AAP, American Academy of Pediatrics; GAS, group A streptococcus; RADT, rapid antigen detection test.

Since the 1980s, rapid antigen detection tests (RADT) are available to facilitate detection of GAS. RADTs provide results within 5 to 10 minutes, do not require any special equipment or personnel, and can be performed at the point of care with a throat swab [6–9]. Guidelines from the AHA, the IDSA, and the AAP recommend the use of RADTs selectively, based on the clinical likelihood of GAS (Table 1) [3–5]. The rationale for selective testing is to increase the rate of positive RADT results, to avoid RADT in children who are more likely to be GAS carriers rather than patients truly infected with GAS, and to contain costs [3, 4, 6].

Controversy persists whether clinical criteria are helpful for selective testing [10–12]. So far, more than ten scoring systems and prediction rules have been proposed to help physicians in expressing the clinical likelihood of GAS in children with pharyngitis [13–15]. We have recently shown that these systems and rules were of limited value, because they were unable to identify patients with low and high probability of GAS who could be managed without RADT [13].

Many previous systems and rules were developed using suboptimal methods [13]. We hypothesized that, using more robust statistical methods, a better performing rule could be developed. Based on data collected in a prospective cohort of 676 French children with pharyngitis, we built a multivariable logistic regression model and derived a decision rule for selective testing from it.

## Materials and methods

### Study design and participants

We used data from a study described in detail elsewhere [13, 16–18]. This was a French prospective multicenter cross-sectional study aimed at assessing the diagnostic accuracy of a RADT (StreptAtest, Dectrapharm, France; a rapid immunochromatographic strip assay) in 678 consenting children aged 3 to 15 years, with throat culture on a blood agar plate in a microbiology laboratory as the reference standard. Seventeen primary care office-based pediatricians participated.

Between October 1, 2010, and May 31, 2011, double throat swabs were collected from consecutive children who had a diagnosis of pharyngitis and had not received antibiotics within the previous week. One swab was used for performing the RADT in the pediatrician’s office following the manufacturer’s instructions; the other swab was sent to the hospital laboratory for throat culture according to standard methods.

Oral parent and patient approval for participation was obtained before inclusion; consent was then written down in the patient’s file. Consent on behalf of the children enrolled was obtained from at least one parent. The study protocol was approved by the Ile-de-France XI institutional review board (no. 09016) and the French administrative authorities (CNIL, no. 1354254; Afssaps, no. 2009-A00086-51).

### Outcome definition and predictor variables

We built a logistic regression model in which the binary outcome of interest was the presence of GAS on throat culture. Based on the literature, the following 17 signs and symptoms were considered as candidate predictor variables: age, sudden onset of sore throat, maximum body temperature (as reported by the accompanying parent), throat pain, cough, rhinorrhea, conjunctivitis, headache, pharyngeal erythema, tonsillar swelling, tonsillar exudate, palatal petechiae, nausea and/or vomiting, abdominal pain, diarrhea, tender cervical lymph nodes, and presence of a scarlatiniform rash (S2 File) [19]. Physicians collecting these signs and symptoms did not know the throat culture results; microbiologists evaluating the throat culture were blinded from clinical information and RADT results.

### Model-based selective testing strategy

**Fig 1:**
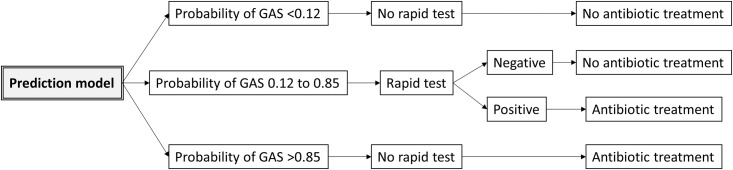
Model-based selective testing strategy. Abbreviations: GAS, group A streptococcus.

After having developed the logistic regression model, we derived the following model-based selective testing strategy (Fig 1):

- low probability of GAS (calculated probability <0.12), no RADT, no antibiotic treatment;
- high probability of GAS (calculated probability >0.85), empiric antibiotic treatment without RADT;
- intermediate probability of GAS (calculated probability between 0.12 and 0.85), RADT for all, antibiotic treatment only with positive result.

The probability cutoffs were chosen because they were used previously in the literature [2, 13]: the 0.12 threshold reflects the average prevalence of GAS pharyngeal carriage in children; the 0.85 threshold corresponds to the minimal positive predictive value of RADTs.

### Statistical analysis

#### Missing data

The number of missing values ranged from 0.4% to 6.2% per candidate predictor variable. Missing values were imputed 10 times using multiple imputations with chained equations as described previously [13]. Statistical analyses were performed separately in each imputed dataset; estimated parameters and corresponding variances were pooled using Rubin’s rule [20]. All statistical analyses were performed in Stata/SE 13 (StataCorp, College Station, Texas).

#### Model building

As recommended, first degree fractional polynomial transformations of the continuous variables age and maximum body temperature were used to explore deviations from linearity in univariable logistic models [21].

All candidate predictor variables were included in the initial multivariable logistic regression model, regardless of their association with the outcome in univariable analysis. Backward stepwise selection of candidate predictor variables was then performed in 200 bootstrap samples, using a *P*-value of 0.157 for removal [22]. The final model retained only predictor variables that were selected in at least 60% of the bootstrap models [23, 24]. We did not add interaction terms in the model, as this may not improve predictions [25]. We estimated associations as odds ratios with corresponding 95% confidence intervals. An odds ratio greater than one indicates an increased risk of GAS within patients with a given sign or symptom, while an odds ratio of less than one indicates a decreased risk of GAS.

#### Model performance

Model performance was assessed in terms of discrimination and calibration. Discrimination was evaluated by calculating the *c*-index, which is equal to the area under the ROC curve. Higher values indicate better performance; a *c*-index of 1 would indicate perfect discrimination. The *c*-index was corrected for optimism using bootstrap internal validation (*b* = 200) [26, 27]. We studied calibration graphically by comparing calculated probabilities of GAS with the observed proportions in groups defined according to quintiles of calculated probabilities.

#### Efficiency and diagnostic accuracy of the model-based strategy

With the final logistic regression model, we calculated the probability of GAS for every child in the study. The distribution of calculated probabilities of GAS was represented graphically using a cumulative distribution function.

The efficiency of a model-based rule for selective testing was expressed as the proportion of participants in whom RADT could be avoided following the rule. We also calculated the sensitivity and specificity of the model-based selective testing strategy. A false positive result was an indication for antibiotics in the absence of GAS; a false negative result, no indication for antibiotics in the presence of GAS.

## Results

### Study participants

One patient with an uninterpretable RADT result and one patient whose throat sample was lost could not be included in the analysis. In the 676 remaining participants there were 313 girls [46%]; the overall mean age was 6.1 [standard deviation, ± 2.5] years. The diagnosis of GAS was confirmed by throat culture in 280 (41.4%; 95% confidence interval [CI], 37.7% to 45.2%). RADT sensitivity was 93% (95% CI, 89% to 95%) for a specificity of 88% (95% CI, 85% to 92%).

### Model development and performance

**Fig 2:**
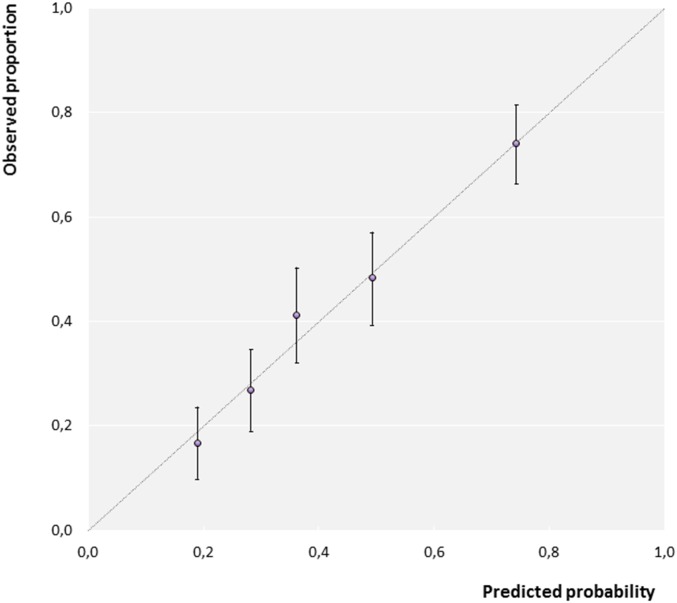
Calibration plot of calculated probabilities of group A streptococcus (GAS) and observed outcomes (N = 676). Circles represent mean calculated probabilities versus observed proportions in subgroups defined by quintiles of the calculated GAS probabilities (*m* = 1). Vertical bars are 95% confidence intervals. Dashed diagonal line represents perfect calibration.

Eight predictor variables were selected in 60% or more of bootstrap samples; these were further retained in the final model. Six variables were significantly associated with GAS, and two were not (Table 2 and Table A in S1 File). Age and maximum body temperature were kept as continuous predictors in the model, after having being scaled and transformed (Table 2). On internal bootstrap validation, the model had an optimism-corrected *c*-index of 0.73. Calibration plots showed good agreement between calculated probabilities of GAS and observed outcomes (Fig 2 and Figure A in S1 File).

**Table 2:** Predictor variables included in the multivariable model.

Predictor variable	Odds ratio (95% CI)	P-value
Age^a^	0.90 (0.84–0.96)	0.003
Temperature^a^	0.99 (0.99–0.99)^b^	0.025
Cough	0.60 (0.40–0.88)	0.009
Rhinorrhea	1.44 (0.98–2.13)	0.065
Palatal petechiae	3.18 (1.99–5.08)	<0.001
Abdominal pain	0.72 (0.50–1.04)	0.077
Tender nodes	2.15 (1.41–3.29)	<0.001
Scarlatiniform rash	9.83 (4.94–19.58)	<0.001

All predictor variables binary coded, except age and maximum body temperature (continuous).

^a^ Predictors were scaled and transformed: Age’ = [(Age/10)^-2^]—2.71 and Temperature’ = Temperature^3^- 58138.

^b^ Exact values: 0.9999476 (0.9999017–0.9999934).

### Efficiency and diagnostic accuracy of the model-based strategy

**Fig 3:**
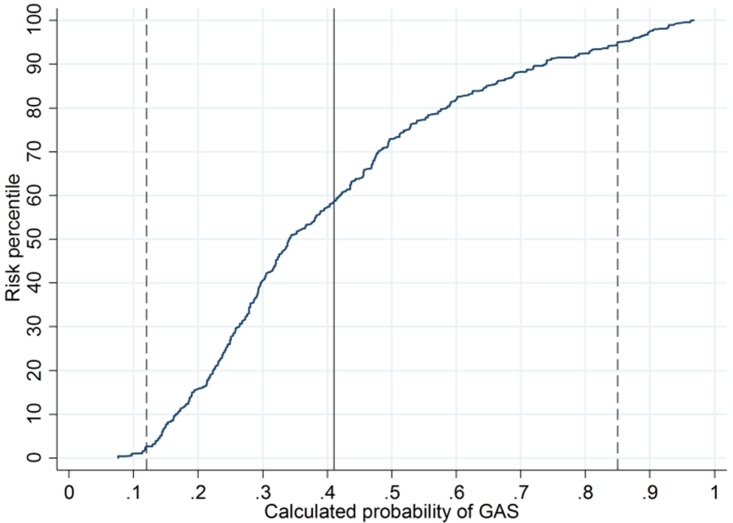
Distribution of calculated probabilities of group A streptococcus (GAS) when applying the clinical prediction model. The vertical dashed lines represent thresholds used to define low and high probability of GAS (calculated probability of GAS<0.12 and >0.85, respectively). The vertical black line represents disease prevalence (0.41).

The calculated probabilities of GAS in our study group ranged from 0.08 to 0.97 across imputed datasets (Fig 3). The proportion of participants with low and high calculated probability of GAS were both <5%. With our model-based rule for selective testing, RADT could be avoided in 6.6% of participants (95% CI, 4.7% to 8.5%). The sensitivity of the model-based selective testing strategy was 92% (95% CI, 89% to 95%) at a specificity of 88% (95% CI, 85% to 91%).

## Discussion

Clinical practice guidelines from the AHA, the IDSA, and the AAP recommend using signs and symptoms to select children with pharyngitis who should undergo RADT for GAS (Table 1) [3–5]. Applying multivariable logistic regression to data from a prospective multicenter cross-sectional study of 676 French children with pharyngitis, we developed and evaluated a model-based rule for selective testing. While the model showed fair discrimination and good calibration, its application would have resulted in a reduction of less than 7% in RADT use. We believe that such a reduction in RADT use is not clinically relevant, considering the additional burden of having to use a computerized model-based decision rule.

Our study has several strengths. First, we included 676 participants, which is larger than most previous studies which aimed at developing prediction models for GAS pharyngitis in children. Second, the prevalence of GAS in our study group was 41%, which is close to that from a recent meta-analysis (37%) [1], and suggests that our cohort resembles other previously published series of children with pharyngitis. Third, we *a priori* defined cutoffs used to consider patients as being at low and high risk of GAS; this may have prevented from bias due data-driven approaches. Finally, we used advanced statistical methods, such as multiple imputations and bootstrap, for a lower risk of bias and optimism-corrected performance estimates.

Our study also has potential limitations. Participants were enrolled by a relatively small sample of seventeen primary care pediatricians, who are part of a research and teaching network (Association Clinique et Thérapeutique Infantile du Val-de-Marne; http://activ-france.fr/), and our results might not be widely generalizable.

We chose the decision thresholds in our model-based rule *a priori*, based on clinical arguments previously reported and used in the literature [2, 13]. Yet we acknowledge that we currently lack agreement on such cutoff values. Guidelines from the AHA and the IDSA only provide lists of signs and symptoms suggestive of GAS or viral etiology, but do not explain how such clinical criteria should be combined for decision making, which leaves primary care physicians with unclear guidance. Consensual decision cutoffs could be identified through a survey involving primary care physicians and infectious diseases specialists.

We used logistic modeling procedures for developing and evaluating our clinical prediction model. We are aware that other modeling approaches exist, such as classification and regression trees, neural networks, and support vector machines [25], but relied on empirical evidence that such techniques may not outperform logistic regression modeling in clinical prediction [28], and that they may be reliable only in the case of very large datasets [29].

A previous systematic review of the literature reported that four of the eight signs and symptoms retained in our model (i.e., cough, palatal petechiae, tender nodes, and scarlatiniform rash) were significantly associated with the presence of GAS in children with pharyngitis [2]; for these variables, the odds ratios that we found were in the same range as those reported previously [2]. However, for two variables in our prediction model (rhinorrhea and abdominal pain), the association with GAS was inverse to that usually reported by other authors [2].

In line with previous findings [13], this study demonstrates that signs and symptoms, though significantly associated with GAS, are not efficient for selective testing. Back in 1954, Breese and Disney studied the accuracy of clinical diagnosis of GAS infection in about 1,200 children with pharyngitis, and concluded that “certain symptoms and signs were suggestive of streptococcal infection but none were diagnostic” [30]. This was later confirmed by many clinical studies, and a meta-analysis of them [2]. Yet the idea persists in several clinical practice guidelines that signs and symptoms are useful for selecting patients who should undergo RADT.

The low efficiency of signs and symptoms in diagnosis of pharyngitis in children may be explained by the relatively weak magnitude of the association between clinical predictor variables and GAS. According to a recent meta-analysis, signs and symptoms have a minimal negative likelihood ratio of 0.4 (for the presence of red tonsils and/or pharynx) and a maximal positive likelihood ratio of 3.9 (for the presence of a scarlatiniform rash) [2]. In comparison, RADTs have, on average, negative and positive likelihood ratios of about 0.15 and 20, respectively [8]. The higher the positive likelihood ratio, the better the test is at ruling in disease; the lower the negative likelihood ratio, the better the test is at ruling out disease. For example, a positive likelihood ratio of 20 means that a positive rapid test result is twenty times more likely in patients with GAS than in patients without GAS. Moreover, clinical variables that are strongly predictive of GAS, such as the presence of a scarlatiniform rash, are unfortunately rarely present in children with pharyngitis.

Based on our findings and the results from a previous external validation study of existing scoring systems and prediction rules [13], we advocate against the use of clinical criteria for selective testing purposes. We believe that the only way to achieve high sensitivity and specificity (about 85% and 95%, respectively) [7–9] for diagnosing GAS is to perform a RADT in all children 3 to 15 years of age presenting with pharyngitis, regardless of their clinical features.

We recommend universal RADT in children with pharyngitis, but some experts argue that this strategy might not be cost-effective [12]. In a recent decision tree analysis, Giraldez-Garcia *et al*, found that a selective testing strategy based on the McIsaac score was more cost-effective than universal RADT [31]. However, they based their calculations on the assumption that the RADT would have a specificity of 78%, which seems particularly low [7–9].

## Conclusions

It might be time to admit that clinical criteria are not useful to predict the presence of GAS and to select pediatric patients who should undergo RADT. Clinical assessment of children with pharyngitis remains crucial to evaluate the presence of complications, such as peritonsillar abscess and acute rheumatic fever.

## Supporting information

S1 File Supporting information. (DOC)

S2 File Minimal dataset. (XLS)

## Abbreviations

CI: confidence interval
GAS: group A streptococcus
RADT: rapid antigen detection test
