# Supplementary material for: Efficiency of a clinical prediction model for selective rapid testing in children with pharyngitis: A prospective, multicenter study
Source: PLoS One. 2017 Feb 24;12(2):e0172871. doi: 10.1371/journal.pone.0172871 (PMC5325561; doi:10.1371/journal.pone.0172871)
Supplement: S1 File — (DOC) [file pone.0172871.s001.doc]

**S1 File. Supporting Information**

Cohen JF, Cohen R, Bidet P, Elbez A, Levy C, Bossuyt P, Chalumeau M. Efficiency of a clinical prediction model for selective rapid testing in children with pharyngitis: A prospective, multicenter study

**Table A. Selection of predictor variables: number of times each predictor was selected in 200 bootstrap backwards stepwise selection procedures, across 10 imputed datasets (N=676).**

Legend: All binary predictor variables, except age and temperature (continuous).

**Figure A. Calibration plots of calculated probabilities of group A streptococcus (GAS) and observed outcomes (N=676).**

Legend: Circles represent mean calculated probabilities versus observed proportions in subgroups defined by quintiles of the calculated GAS probabilities (m=2 to m=9). Vertical bars are 95% confidence intervals. Dashed diagonal line represents perfect calibration.

This supplementary material is provided by the authors to give readers additional information about their work.

**Table A**. Selection of predictor variables: number of times each predictor was selected in 200 bootstrap backwards stepwise selection procedures, across 10 imputed datasets (N=676).

|  | | **Imputed dataset** | | | | | | | | | |  |  |
| --- | --- | --- | --- | --- | --- | --- | --- | --- | --- | --- | --- | --- | --- |
|  | | **1** | **2** | **3** | **4** | **5** | **6** | **7** | **8** | **9** | **10** | **Total (%)** | **In final model** |
| **Candidate predictor** | |  |  |  |  |  |  |  |  |  |  |  |  |
|  | Age, years | 187 | 190 | 189 | 189 | 177 | 190 | 188 | 185 | 185 | 188 | 1868 (93.4) | **Yes** |
|  | Sudden onset | 44 | 43 | 39 | 45 | 33 | 34 | 39 | 34 | 42 | 38 | 391 (19.6) | No |
|  | Body temperature | 163 | 151 | 159 | 152 | 142 | 155 | 158 | 156 | 151 | 153 | 1540 (77.0) | **Yes** |
|  | Throat pain | 68 | 43 | 88 | 66 | 112 | 95 | 77 | 52 | 55 | 63 | 719 (36.0) | No |
|  | Cough | 165 | 177 | 181 | 179 | 179 | 179 | 185 | 172 | 183 | 177 | 1777 (88.9) | **Yes** |
|  | Rhinorrhea | 112 | 120 | 128 | 130 | 134 | 137 | 148 | 128 | 144 | 139 | 1320 (66.0) | **Yes** |
|  | Conjunctivitis | 65 | 71 | 65 | 64 | 56 | 54 | 63 | 59 | 60 | 58 | 615 (30.8) | No |
|  | Headache | 45 | 37 | 53 | 22 | 51 | 35 | 29 | 55 | 43 | 51 | 421 (21.1) | No |
|  | Pharyngeal erythema | 30 | 40 | 47 | 45 | 38 | 35 | 38 | 40 | 33 | 34 | 380 (19.0) | No |
|  | Tonsillar swelling | 74 | 75 | 74 | 102 | 70 | 74 | 110 | 83 | 49 | 63 | 774 (38.7) | No |
|  | Tonsillar exudate | 108 | 127 | 88 | 138 | 114 | 97 | 97 | 99 | 118 | 104 | 1090 (54.5) | No |
|  | Palatal petechiae | 200 | 200 | 200 | 200 | 200 | 199 | 200 | 199 | 200 | 200 | 1998 (99.9) | **Yes** |
|  | Nausea/vomiting | 101 | 112 | 100 | 102 | 108 | 118 | 105 | 85 | 81 | 94 | 1006 (50.3) | No |
|  | Abdominal pain | 159 | 152 | 151 | 157 | 146 | 136 | 143 | 130 | 127 | 130 | 1431 (71.6) | **Yes** |
|  | Diarrhea | 75 | 86 | 82 | 47 | 62 | 76 | 92 | 90 | 72 | 106 | 788 (39.4) | No |
|  | Tender cervical nodes | 191 | 193 | 197 | 193 | 196 | 195 | 195 | 196 | 197 | 188 | 1941 (97.1) | **Yes** |
|  | Scarlatiniform rash | 200 | 200 | 200 | 200 | 200 | 200 | 200 | 200 | 200 | 200 | 2000 (100) | **Yes** |
| ***C-*index final model** | | 0.737 | 0.737 | 0.742 | 0.744 | 0.741 | 0.737 | 0.743 | 0.740 | 0.737 | 0.736 | n/a | n/a |
| **Optimism** | | 0.013 | 0.013 | 0.013 | 0.013 | 0.013 | 0.014 | 0.013 | 0.013 | 0.013 | 0.014 | n/a | n/a |

**Figure A. Calibration plots of calculated probabilities of group A streptococcus (GAS) and observed outcomes (N=676).**
